# Value of Eye-Tracking Data for Classification of Information Processing–Intensive Handling Tasks: Quasi-Experimental Study on Cognition and User Interface Design

**DOI:** 10.2196/15581

**Published:** 2020-06-03

**Authors:** Stephan Wegner, Quentin Lohmeyer, Dimitri Wahlen, Sandra Neumann, Jean-Claude Groebli, Mirko Meboldt

**Affiliations:** 1 Product Development Group Zurich, Institute of Design, Materials and Fabrication Department of Mechanical and Process Engineering Swiss Federal Institute of Technology in Zurich Zürich Switzerland; 2 Peripal AG Zürich Switzerland

**Keywords:** human factors engineering, mobile eye tracking, benchmarking, home care, usability, self-management, quantitative research, quantitative evaluation

## Abstract

**Background:**

In order to give a wide range of people the opportunity to ensure and support home care, one approach is to develop medical devices that are as user-friendly as possible. This allows nonexperts to use medical devices that were originally too complicated to use. For a user-centric development of such medical devices, it is essential to understand which user interface design best supports patients, caregivers, and health care professionals.

**Objective:**

Using the benefits of mobile eye tracking, this work aims to gain a deeper understanding of the challenges of user cognition. As a consequence, its goal is to identify the obstacles to the usability of the features of two different designs of a single medical device user interface. The medical device is a patient assistance device for home use in peritoneal dialysis therapy.

**Methods:**

A total of 16 participants, with a subset of seniors (8/16, mean age 73.7 years) and young adults (8/16, mean age 25.0 years), were recruited and participated in this study. The handling cycle consisted of seven main tasks. Data analysis started with the analysis of task effectiveness for searching for error-related tasks. Subsequently, the in-depth gaze data analysis focused on these identified critical tasks. In order to understand the challenges of user cognition in critical tasks, gaze data were analyzed with respect to individual user interface features of the medical device system. Therefore, it focused on the two dimensions of dwell time and fixation duration of the gaze.

**Results:**

In total, 97% of the handling steps for design 1 and 96% for design 2 were performed correctly, with the main challenges being task 1 insert, task 2 connect, and task 6 disconnect for both designs. In order to understand the two analyzed dimensions of the physiological measurements simultaneously, the authors propose a new graphical representation. It distinguishes four different patterns to compare the eye movements associated with the two designs. The patterns identified for the critical tasks are consistent with the results of the task performance.

**Conclusions:**

This study showed that mobile eye tracking provides insights into information processing in intensive handling tasks related to individual user interface features. The evaluation of each feature of the user interface promises an optimal design by combining the best found features. In this way, manufacturers are able to develop products that can be used by untrained people without prior knowledge. This would allow home care to be provided not only by highly qualified nurses and caregivers, but also by patients themselves, partners, children, or neighbors.

## Introduction

Chronically ill patients cared for at home experience a higher health-related quality of life and a normalization of everyday life that is less dominated by the disease [1-3]. Therefore, 82% of end-stage renal disease patients and their families, if fully informed about their treatment options, would choose a home modality [4]. However, only 14% of dialysis patients in Europe are treated at home [5]. The main obstacle to home care is the availability of caregivers such as community nurses, neighbors, or relatives [6,7]. In order to allow a broad range of people the opportunity to ensure and support home care, one approach is to design medical devices with greater ease of use. This allows nonexperts to use medical devices that were originally too complicated to use. For user-centric development of such medical devices, it is essential to understand which user interface (UI) designs best support patients, caregivers, and health care professionals [8,9].

Human factors engineering drives user-oriented design and must test customized product UIs with intended users to determine the ideal level of mental workload. According to Kantowitz [10], mental workload is a subset of attention and the link between the demands of the environment and the capacity of the organism; it cannot be directly assessed. In a usability evaluation, the abstract term demand of the environment means fulfilling a task correctly. Consequently, when use errors occur, demand has not been met, and mental workload may have been too high or too low. This may be evaluated by analyzing the distribution and characteristic of attention in use error–related tasks.

Methods such as observations, questionnaires, and interviews are used to gain insight into the usability of an interface, but the focus is mainly on the graphical UI on a screen [9,11-15]. However, users gain most information through visual perception [16], and the short-term memory has only a limited capacity [17,18]. Therefore, it is difficult to understand the causes of use errors using traditional methods only.

Eye tracking provides a first-person perspective of the user and continuous localization of the gaze point. According to Hoang Duc et al [19], “tracking eye movements has the potential to provide a more direct measure of where attention is deployed since the direction of gaze is generally considered to be tightly coupled to the orienting of attention.” Furthermore, “when people attend to a particular spatial location, there is greater neural processing in portions of the visual cortex corresponding to that location” [20]. Eye tracking thus allows objective feedback to find perception problems [21,22] and gain valuable insights into hotspots in attention distribution on the UI. This information can be used for both qualitative and quantitative evaluation of the usability of the UI. As a result, in recent years eye tracking has increasingly become a method for testing attention and improving or evaluating the features of UIs. Examples are web and print advertisements [23,24] and graphical representations like x-ray images of patients [25]. More complex subjects of the investigations include graphical UIs such as computer tomography interfaces [26] or spacecraft displays [27,28]. Further, there are single studies where eye tracking is used to evaluate highly interactive UIs of tangible products like smart TVs [29], smartwatches [12,21], or medical devices [30,31].

Most studies used a remote eye-tracking system where the stimulus is presented on a screen and participants are asked to sit still in front of a desk. Aside from this setup, mobile eye tracking with minimally invasive head-mounted systems provides a degree of freedom in movability. This promises natural user behavior in the testing of tangible medical devices [30].

In a first step of the eye-tracking data analysis, the raw gaze point data are classified into three events: fixation (nearly no eye movement), saccade (fast eye movement), and blink (closed eye). Since classified gaze data contain no semantic information on the looked-at objects or features, a second step of areas of interest mapping is needed. In this step, the single fixation events are manually assigned to the specific looked-at objects or UI features. As a result, data can subsequently be analyzed object-related in terms of durations of single fixations or cumulative dwell times (DTs) on an object or feature for a particular task. Fixation duration (FD), describing a property of visual attention per unit, is associated with the processing depth, which when increased leads to longer fixations [32-35], and with the rate of information extraction [23,35,36]. DT, describing the sum of visual attention related to specific objects or features, is associated with the length of the information extraction [28,37]. Thus, these measurements represent attention and, in the context of handling tasks, mental workload as a subset of attention in two dimensions.

Using the benefits of mobile eye tracking, we aimed to gain a deeper understanding of the challenges and differences in user cognition and thus identify obstacles to the user-friendliness of single UI features of a patient assistance device intended for home use in peritoneal dialysis (PD) therapy. This paper describes, to our knowledge, the first benchmark tests of two different UI designs based on physiological measurements using mobile eye tracking. The underlying research questions of this work are as follows:

- RQ1: Do slight differences in the design of the UI of a patient assistant device lead to differences in the effectiveness of use?
- RQ2: What are the differences in visual perception between two UI designs of a patient assistant device related to single task-relevant UI features in use error–related handling tasks?

## Methods

### Summary

The aim of this work was to gain a deeper understanding of the challenges in user cognition and thus the obstacles to user-friendliness of single UI features of a medical device. Therefore, a quasi-experimental study was conducted for data collection with the medical device with the intention of being as realistic as possible and representing the intended use. As a result, naïve representatives of the user group of patients (young adults and seniors) were recruited, and the study was conducted in the intended environment.

### Stimuli

**Figure 1:**
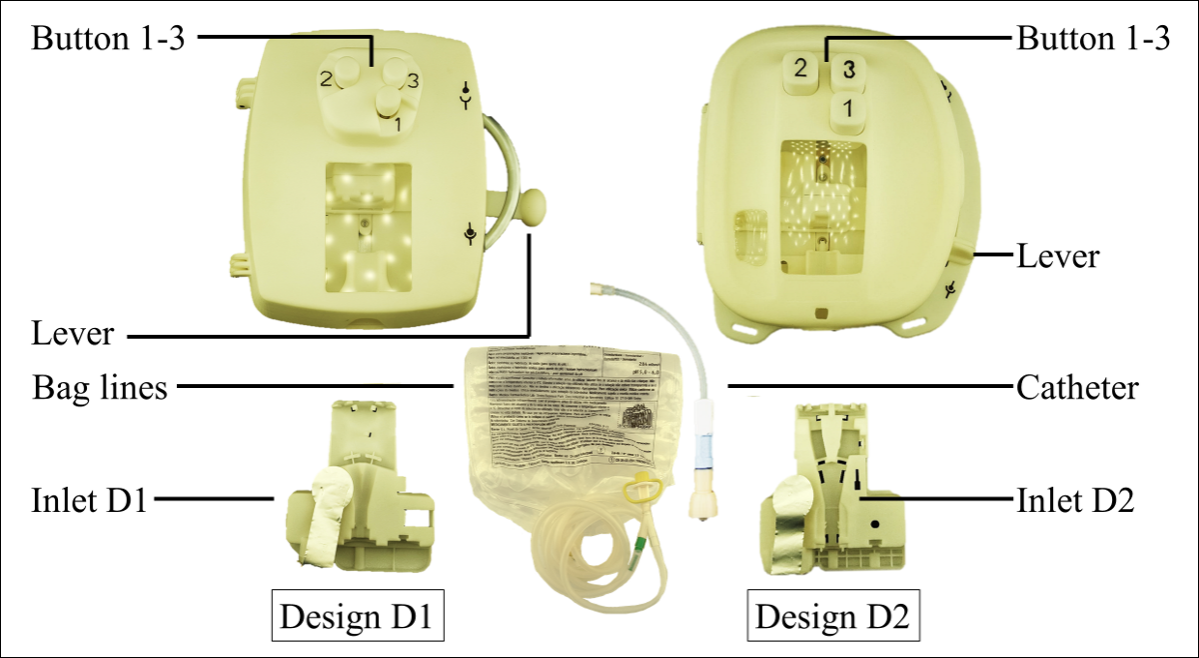
Illustration of user interface designs D1 (left) and D2 (right) including features lever and buttons 1-3. Additional parts for the therapy handling with the medical device are bag lines and catheter (standard parts used in therapy) and inlets D1 and D2, compatible with their respective designs.

The stimuli of the study were prototypes with two different UI designs (D1 and D2, see Figure 1) of a medical device system. The system consists of medical device, inlet for guiding and manipulating a bag system with dialysis fluid, and catheter, which is connected to the patient in the real therapy application. The most important interface features of the medical device are the buttons for manipulating the bag system and the lever for moving the inlet inside the device. The inlet has functions for fixing, clamping, and opening a predetermined breaking point feature inside the bag lines. The medical device system supports PD handling and is aimed at adults aged 18 and older. The stimuli provide acoustic (click sounds), haptic (positioning by stops), and visual (clear states and observation windows) feedback. Both prototypes support the same functionalities and require the same handling steps. At the top level, appearance of the UI designs was neutral in a monochrome design, as shown in Figure 1, to eliminate the effects of different coloring as an additional variable.

### Recruitment and Data Exclusion

A total of 25 participants (18 men and 7 women, average 50.2 years, range 24 to 90 years) were recruited and participated in this study. The sample was recruited from a retirement home (10 men and 5 women, average 74.0 years, range 67 to 90 years) and from university (8 men and 2 women, average 25.1 years, range 24 to 26 years). In the PD patient population in Europe, 52% are younger than age 65 years [5]. Due to potential technical challenges with the eye-tracking technology related to the physiology of the eye area, which is especially relevant for seniors as reported by Bojko [38], more participants were invited than analyzed in the final analysis. All participants were in good physical and mental condition and assessed the suitability of study participation themselves. No participant was familiar with PD therapy or mobile eye tracking. All participants had normal or corrected vision with contact or corrective lenses that could be connected to the mobile eye-tracking system.

One senior left the study prematurely after the first handling cycle and was therefore excluded from the analysis. For five seniors and one young adult, data quality was insufficient due to measure errors by the eye tracker resulting from drooping eyelids, watery eyes, or long eyelashes. In order to achieve a counterbalance in terms of the order of use of the two designs and represent the target population characteristic of PD patients in age, the data sets of a randomly selected senior and young adult were not included in the data analysis. Thus, a total of 16 data sets with 8 data sets from each group of young adults (25.0 years on average) and seniors (73.7 years on average) could be analyzed. Four participants in each group started with D1 and four with D2, achieving a complete counterbalance. As a result, this within-subject design mitigated the effect of individuality. Consequently, measures that naturally differ from participant to participant, such as FDs, could be compared with this balanced design of the study.

### Study Procedure

**Figure 2:**
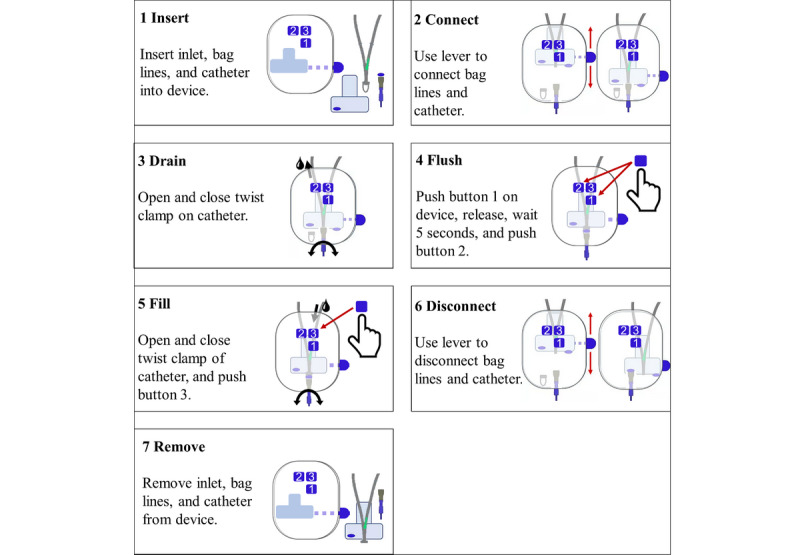
Seven tasks in medical device handling cycle. User interacts manually with inlet, bag lines, catheter, and user interface features lever and buttons 1-3.

When participants arrived in the test environment, they were welcomed and thanked for their participation. Before the study began, participants were asked to read information on the goal of the study, data safety, and data management. If they agreed to participate in the study, they were asked to sign the consent form. Subsequently, participants put on the mobile eye-tracking system, and the moderator conducted a 3-point calibration. Since all participants were beginners in PD therapy and in the use of the device, the moderator briefly described the disease and associated PD therapy. Next, the moderator demonstrated the handling procedure with a low-level representation of the UI, designed and built for this purpose, and the devices. After the introduction, participants performed the handling cycle of tasks 1 through 7 in a simulated PD therapy (see Figure 2), starting either with D1 or D2 and guided by written instructions. Each instruction was printed in a neutral design on an individual sheet to test the usability of the medical device and not the instruction. There was no time limit for the fulfillment of tasks, and the moderator assisted only in cases where the study would otherwise have had to be terminated due to the use error. Subsequent to the first completed handling cycle, participants were asked to give their feedback on usability in a semistructured interview with predefined high-level questions asking for general feedback on tasks related to use errors, guiding to the root causes of handling difficulties and use errors. Starting with the handling cycle, this process was repeated for the remaining prototype of the UI design.

### Data Analysis

In the data analysis, a 2-step approach was used. It started with the analysis of task effectivity searching for use error–related tasks. Subsequently, the in-depth gaze data analysis focused on these identified critical tasks.

For analysis of the task effectivity, the handling process of participants was observed via a live recording from the first person’s perspective from the eye-tracking system. The performance in each task was evaluated by an observer. In the evaluation, two categories were distinguished according to the international standard IEC 62366-1 (2015). The first category, safe use, is defined as “normal use without use error” [39]. The second category, use error, is defined as “user action or lack of user action while using the medical device that leads to a different result than that intended by the manufacturer or expected by the user” [39].

Gaze data were recorded with the mobile eye-tracking system, SMI Eye Tracking Glasses 2 (SensoMotoric Instruments GmbH), with a scene resolution of 1280×960 pixels (viewing angle: 60° horizontal, 46° vertical) of the front camera offering a sampling frequency of 24 Hz with the gaze point measurement having an accuracy of 0.5° over all distances. The raw gaze point data were classified into the events of fixations, saccades, and blinks by SMI BeGaze version 3.7 (SensoMotoric Instruments GmbH). Subsequent to the areas of interest analysis, information on FD for depth and rate and DT for length of information extraction related to particular objects and features of the medical device system (Figure 1) were calculated. This information was used to understand the challenges of user cognition in use error–related tasks. Blinks were not considered in this work.

Combining the information on FD and DT, the data were analyzed with a multivariate analysis of variance (MANOVA) using SPSS Statistics 24 (IBM Corp). The MANOVA had one independent variable with two levels, D1 and D2 (see Figure 1), two dependent variables, FD and DT, both measured on a ordinal level and representing the rank of the mean measurements for every participant for the UI features in error-related tasks.

**Figure 3:**
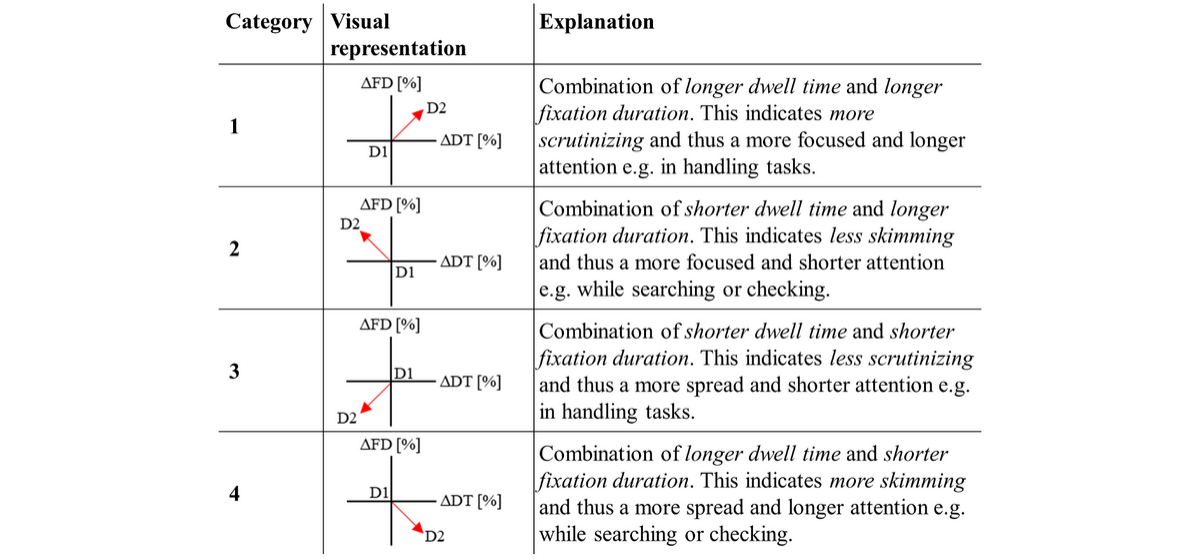
Visualization of shifts in two dimensions of the physiological gaze data measurements fixation duration and dwell time. The displayed shifts are from a Design D1 in the coordinate origin to a Design D2, presented in the middle column. In total, a distinction is made between the four categories. The right column explains the four patterns.

**Figure 4:**
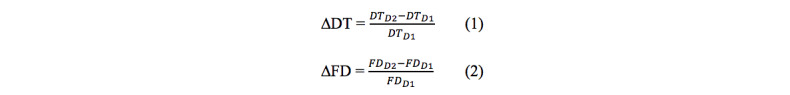
Equations for calculating the values of the shift in both dimensions of dwell time and fixation duration (∆DT and ∆FD) from UI design D1 to D2.

For a better understanding of the two analyzed dimensions, length and depth of visual perception, Figure 3 combines information on the two measured parameters. Evaluating user perception of all participants as a whole, it shows the relationship between mean FD and mean DT for individual UI features of D1 compared with D2. Based on the two analyzed dimensions assigned to D1 in the coordinate origin, the mean FD and mean DT of D2 can be longer or shorter. Consequently, four different categories or patterns can be distinguished. A suggested interpretation of these patterns in terms of workload or gaze behavior is shown in Figure 3. Equations for calculating the values of the shift in both dimensions (∆DT and ∆FD) from D1 to D2 for the diagram can be seen in Figure 4.

## Results

**Figure 5:**
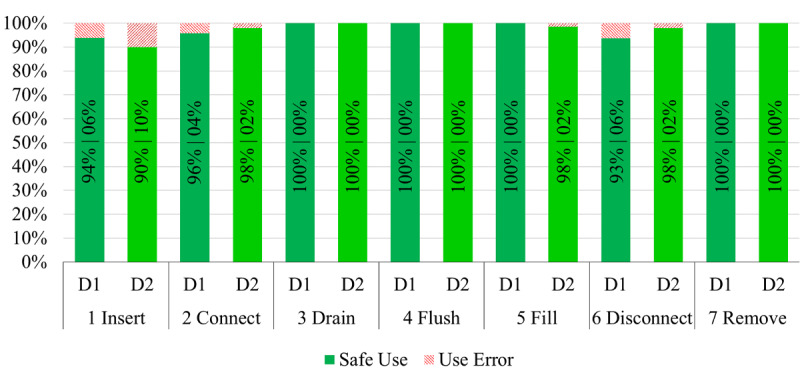
Comparison of task performance between D1 and D2 for all seven tasks. Evaluation in two categories, safe use and use error, according to International Electrotechnical Commission 62366-1 (2015).

Each of the 16 participants performed 30 handling steps in the 7 tasks with both UI designs, resulting in 480 evaluated handling steps for each UI design. The results of the task performance are shown in Figure 5. Overall, 97% of the handling steps were performed correctly for D1 and 96% for D2.

According to the results, the main challenges were in task 1 (insert), task 2 (connect), and task 6 (disconnect) for both UI designs. The remaining four handling tasks were performed without errors, except for one missing catheter closure in task 5 (fill) with D2. Observed use errors in the first task were mainly incorrectly inserted bag lines in the inlet. Further use errors were forgetting to attach the cap of the bag lines to a safety feature on the device and folding the protective film of the inlet outwards. All use errors were discovered and corrected by participants at a later stage of the handling cycle. In task 2 (connect), use errors occurred when the lever should have been used to connect bag lines and catheter. In task 6 (disconnect), some participants forgot to operate the lever for disconnecting catheter from bag lines and for placing a new cap onto catheter. In the semistructured interview, participants mentioned difficulties positioning bag lines and catheter in the inlet, oblivion of some details in the handling from the presentation, hesitation because of fear of breaking something, and misleading wording in the instructions for tasks 2 (connect) and 6 (disconnect). In addition, participants gave positive and negative feedback on the overall impression and experience with the device.

Multimedia Appendix 1 focuses on handling tasks with observed use errors and shows the results of the data analysis of the physiological gaze data in both dimensions. The mean values for FD are given in milliseconds and for DT in seconds. The mean FD for single UI features was between 149 and 405 milliseconds. The mean DT for single UI features was between 0.3 and 28 seconds. At task 1 (insert), there were large shifts from the UI features bag lines, inlet, and catheter to the UI features levers and buttons. While the first group had average DTs between 7 and 28 seconds, the second group had average DTs between 0.3 and 3 seconds. At task 2 (connect) and task 6 (disconnect), the DT varied from less than 1 second for the buttons to 3 seconds for bag lines and catheter. For the mean FD, clustering was not found in any of the three tasks.

A MANOVA revealed statistically significant differences between D1 and D2 for catheter (Pillai trace=.216, F_2,29_=3.985, *P*=.03) and for lever (Pillai trace=.348, F_2,25_=6.674, *P*=.005) in task 1 (insert) and for inlet (Pillai trace=.22, F_2,25_=3.534, *P*=.045) in task 6 (disconnect). All other UI features showed no statistically significant differences in the three error-related tasks.

**Figure 6:**
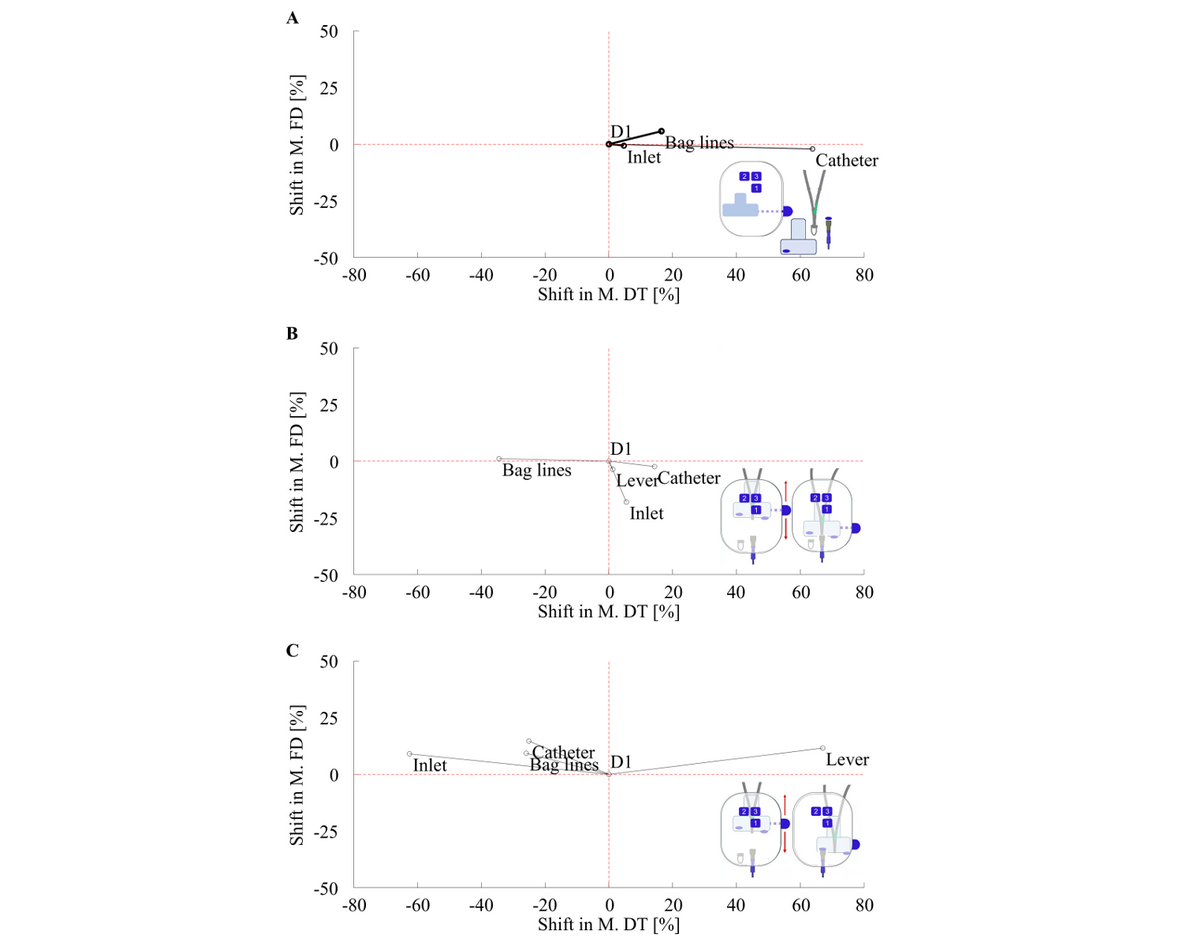
Shifts from D1 (in the coordinate origin) to D2 in terms of mean fixation duration (ordinate) and mean dwell time (abscissa) for task 1 insert (A), task 2 connect (B), and task 6 disconnect (C). The relevant user interface features in these three tasks are bag lines, inlet, catheter, and lever.

For better understanding, Figure 6 visualizes the data presented in Multimedia Appendix 1. As shown in Figure 3, this visualization combines FD and DT as two dimensions of the gaze data. In task 1 (insert; Figure 6A), the mean DT for all task-relevant UI features is longer for D2. The bag lines show a strong category 1 pattern, while the other two UI features show little to no shift for the mean FD. For task 2 (connect; Figure 6B), three UI features show a strong category 4 pattern, while the bag lines show mainly shorter mean DTs and only a slightly longer mean FD, thus showing a weak category 2 pattern. For task 6 (disconnect; Figure 6C), the UI elements located inside the device in this task show a strong category 2 pattern, while the lever on the outside of the device shows a strong category 1 pattern.

## Discussion

### Principal Findings

Task performance analysis generally showed little or no use errors in the various handling tasks for both UI designs (see Figure 5). The tasks with observed use errors were the insertion of material and connection and disconnection of bag lines and catheter. In line with the observations, participants described in semistructured interviews difficulties in the execution and in remembering of the correct handling step details in the observed use error–related tasks. Further, they reported misleading wording in the instructions as the explanation for their use errors in task 2 (connect) and task 6 (disconnect), thus providing additional information for the development of the supplementary material. In the first task, most use errors occurred when inserting the bag lines into the inlet. For this task, Figure 6A shows a category 1 pattern with longer mean DT and longer mean FD for the bag lines. Therefore, the results of gaze data analysis are consistent with the results of task performance. Gaze data shows more scrutinizing for D2 compared with D1 in order to insert bag lines and catheter into inlet and device. The longer and higher depth in visual perception indicates a higher mental workload for this task using D2.

When connecting and disconnecting the catheter, some participants missed pulling down the lever to connect bag lines and catheter again and putting a new cap on the catheter. For connecting and disconnecting bag lines and catheter, the most important interface features show category 4 patterns (task 2, Figure 6B) and category 2 patterns (task 6, Figure 6C). This visual pattern indicates more skimming behavior for task 2 and less skimming behavior for task 6. This in turn indicates more visual controls when connecting bag lines and catheter in task 2 for D2. Compared with task performance, this seems to result in slightly fewer use errors for D2 (2% vs 4%). For task 6, results indicate less visual searching associated with the relevant features inlet and catheter for D2 when a new cap is placed on the catheter. In a comparison of the two UI designs, the main difference between D1 and D2 is the position of the top window. With D2, the user can better see the inlet. This may help finding the important features while a new cap is placed on the catheter. Furthermore, the lever in task 6 shows a category 1 pattern associated with a longer and higher depth in visual perception for D2. Although the results show fewer use errors, handling the lever with D2 appears to be mentally more difficult than with D1.

When evaluating the total mental workload of the medical device system, the analyzed UI features of the medical device showed shifts in both the mean FD and mean DT. The mean FD varied from 149 to 405 milliseconds in the critical tasks across all features (Multimedia Appendix 1). In order to be able to interpret these values, the results of three different task examples as described in the literature are compared. In a case study of a driving situation described by Velichkovsky et al [34], the values for the mean FD were between 499 and 543 milliseconds. Bojko et al [33] reported in an evaluation of drug label designs that the FD varied between 260 and 392 milliseconds. Just and Carpenter [35] observed a mean FD of 477 milliseconds observing the task of reading a scientific text. Compared with these studies, the mean FD of the handling cycle is in the same range as reading a drug label. The mean DT in the critical tasks varied in a range from 0.3 to 28.3 seconds (Multimedia Appendix 1). Especially in the first task, the insertion of the material in both UI designs required longer DT for bag lines and catheter compared with other tasks. This shows that this task requires special attention from the user. This is supported by significant differences in a MANOVA for the catheter in the considered task. The statistical analysis showed only in two other cases significant differences in the gaze data. The reason for merely three significant differences is probably because of the low level of variation in the design.

Based on the results of this study, benchmarking D1 and D2 showed the following. Inserting the material seemed to be challenging for both UI designs in general. Therefore, the guiding material (manual and quick starting guide) and training should focus on this task. The lever of D1 seemed to result in lower mental workload. It has a more dominant appearance compared with D2, where the lever is integrated into the housing for protection in case of a fall. The UI design D2 of the inlet seems to be easier to perceive visually. The higher position of the top window in D2 shows a positive impact on the task connecting and disconnecting bag lines and catheter.

Analysis of two dimensions of visual perception using eye tracking provided a detailed picture of the length and depth of the visual perception and therefore the challenges in user cognition and ease of use. Results highlighted the differences in information extraction for different UI features in single tasks. This information helped human factors engineering to focus the development on the critical UI features. Following this work, a summative study evaluated the final UI of the device. This final design and the instructions incorporated the results of this study, such as the detailed description of the insertion of the material and the coloring of the main UI features to guide the user’s gaze. The summative study included patients, relatives, nurses, and physicians. They represented the later user population in the characteristic in age, preknowledge, and comorbidities. Patients had two types of comorbidities, such as arthritis and Reynaud syndrome, in addition to the renal disease with its own accompanying symptoms. The summative study confirmed the safety, efficiency, and effectiveness of use [40].

### Limitations

Due to the novelty of the medical device presented in this study, there are several limitations regarding the results. First, participants were not patients in the real therapy. They were beginners who had no experience in this specific therapy or associated tasks. Furthermore, the device was not used in the real therapy application but in a simulation. These factors provide information on how forgetfulness or even dementia would influence use of the medical device in the later use by patients. Second, when the final product is used, individual training of the user is mandatory and labeling material supports the user. This support was not provided in this study. Instead, a presentation with an additional low-level representation of the UI and a neutral text of the seven tasks guided participants through the handling cycle. Consequently, the focus was on intuitive task performance and perception of information depending on the different UI designs. Third, the design of the two different top-level designs was similar due to a unicolored representation. This is not a strong contrast between the main UI functions and the rest of the medical device. As stated in Methods, this was chosen to eliminate influences of different coloring as an additional influencing factor. At the level of gaze data analysis, representation of the combination of mean FD and mean DT is the first published. Further research is needed to assess whether identified patterns apply to different usability studies with different tasks and stimuli.

### Conclusion

The prototypes of the medical device system as stimuli of the study had only little differences in the single UI features. Consequently, results in the effectiveness of use revealed only marginal differences, with a maximum of 6% versus 10% use errors in task 1 (insert). Based on the two dimensions of the physiological gaze data measurements FD and DT, four distinct patterns could be distinguished between the two UI designs. A MANOVA revealed statistically significant differences in these patterns for three UI features.

Studying the impact on the usability of alternatives of different UI designs is crucial to understand which best supports the user. Traditional methods such as observation, interviews, or questionnaires tend to give feedback only at the level of the UI as a whole. Furthermore, when it comes to reporting usability issues or first impressions of the medical device during interviews or questionnaires, several challenges arise. Test participants may forget to report their impressions or adapt their answers to social expectations [30,41]. This makes it difficult to identify the root causes of usability problems and thus the necessary changes in UI design. In alignment with Lohmeyer et al [31] and Koester et al [30], this study showed that mobile eye tracking provides objective quantitative results based on physiological measurements related to individual UI features. These results can be used to evaluate usability in much more detail compared with traditional methods.

This information is crucial to be able to adapt the design of a product to the needs of the users. Therefore, results of usability testing must be more detailed than just a yes-or-no result of use errors. On the contrary, evaluation of each feature of the UI promises to achieve the best possible UI design by combining the best features found. This combined solution would therefore offer the highest level of usability. In this way, manufacturers can develop products that can be used even by untrained people without prior knowledge. This would allow home care to be provided not only by highly qualified nurses and caregivers, but also by patients themselves, partners, children, or neighbors. This would contribute to removing barriers to home care and thus to a higher quality of life and normalization of everyday life, which is less dominated by illness for patients.

## Appendix

Multimedia Appendix 1 Analysis of eye tracking metrics for user interface features bag lines, inlet, catheter, lever, and buttons. Mean fixation duration (FD) in milliseconds and mean dwell time (DT) of the gaze in seconds for user interface designs D1 and D2. Evaluated tasks are task 1 (insert), task 2 (connect), and task 6 (disconnect). Multivariate analysis of variance analyzed the combination of FD and DT for significant differences according to the Pillai trace (p) between D1 and D2.

## Abbreviations

DT: dwell time
FD: fixation duration
MANOVA: multivariate analysis of variance
PD: peritoneal dialysis
UI: user interface
