# Supplementary material for: Value of Eye-Tracking Data for Classification of Information Processing–Intensive Handling Tasks: Quasi-Experimental Study on Cognition and User Interface Design
Source: JMIR Hum Factors. 2020 Jun 3;7(2):e15581. doi: 10.2196/15581 (PMC7301256; doi:10.2196/15581)
Supplement: Multimedia Appendix 1 [file humanfactors_v7i2e15581_app1.docx]

Multimedia Appendix 1. Analysis of eye tracking metrics for user interface features bag lines, inlet, catheter, lever, and buttons. Mean fixation duration (FD) in milliseconds and mean dwell time (DT) of the gaze in seconds for user interface designs D1 and D2. Evaluated tasks are task 1 insert, task 2 connect, and task 6 disconnect. Multivariate analysis of variance analyzed the combination of FD and DT for significant differences according to a Pillai trace (p) between D1 and D2.

|  | User interface features | | | | | | | | | |
| --- | --- | --- | --- | --- | --- | --- | --- | --- | --- | --- |
|  | Bag lines | | Inlet | | Catheter | | Lever | | Buttons | |
|  | FD^a^ | DT^b^ | FD | DT | FD | DT | FD | DT | FD | DT |
|  |  | |  | |  | |  | |  | |
| **Task 1** |  | |  | |  | |  | |  | |
| D1 | 383 | 24.3 | 309 | 19.2 | 334 | 7.4 | 239 | 1.0 | 268 | 0.5 |
| D2 | 405 | 28.3 | 307 | 20.1 | 327 | 12.1 | 337 | 2.8 | 223 | 0.6 |
| p^c^ | 0.311 | | 0.891 | | 0.030 | | 0.005 | | 0.435 | |
| **Task 2** |  | |  | |  | |  | |  | |
| D1 | 356 | 2.7 | 349 | 1. 5 | 380 | 2.5 | 309 | 1.7 | 236 | 0.3 |
| D2 | 360 | 1.8 | 286 | 1.5 | 371 | 2.8 | 298 | 1.7 | 195 | 1.0 |
| p | 0.473 | | 0.471 | | 0.393 | | 0.943 | | 0.799 | |
| **Task 6** |  | |  | |  | |  | |  | |
| D1 | 333 | 1.0 | 342 | 1. 5 | 299 | 2.2 | 259 | 1.0 | 199 | 0.3 |
| D2 | 364 | 0. 8 | 373 | 1.6 | 343 | 1.7 | 289 | 1.6 | 149 | 0.4 |
| p | 0.667 | | 0.045 | | 0.703 | | 0.846 | | 0.962 | |

^a^FD: fixation duration.

^b^DT: dwell time.

^c^p: Pillai trace.
